# Upadacitinib in active non-radiographic axial spondyloarthritis: 2-year data from the phase 3 SELECT-AXIS 2 study

**DOI:** 10.1186/s13075-024-03441-3

**Published:** 2025-02-04

**Authors:** Filip Van den Bosch, Atul Deodhar, Denis Poddubnyy, Walter P. Maksymowych, Désirée van der Heijde, Tae-Hwan Kim, Mitsumasa Kishimoto, Xenofon Baraliakos, Xianwei Bu, Ivan Lagunes-Galindo, In-Ho Song, Peter Wung, Koji Kato, Anna Shmagel

**Affiliations:** 1 Department of Internal Medicine and Pediatrics, VIB Center for Inflammation Research, Ghent University, Ghent, Belgium; 2 Division of Arthritis & Rheumatic Diseases, Oregon Health & Science University, Portland, OR USA; 3 Department of Gastroenterology, Infectious Diseases and Rheumatology, Charité Universitätsmedizin, Berlin, Germany; 4 Department of Medicine, University of Alberta, Edmonton, AB Canada; 5 Department of Rheumatology, Leiden University Medical Center, Leiden, The Netherlands; 6 Department of Rheumatology, Hanyang University Hospital for Rheumatic Diseases, Seoul, Republic of Korea; 7 Department of Nephrology and Rheumatology, Kyorin University School of Medicine, Tokyo, Japan; 8 Rheumazentrum Ruhrgebiet Herne, Ruhr-University Bochum, Herne, Germany; 9 AbbVie Inc, North Chicago, IL USA

**Keywords:** Axial spondyloarthritis, Disease activity, Inflammation, Janus kinase inhibitor, Safety, Upadacitinib

## Abstract

**Background:**

In SELECT-AXIS 2, upadacitinib improved the signs and symptoms of active non-radiographic axial spondyloarthritis (nr-axSpA) through 52 weeks versus placebo and was well tolerated. Here, we evaluated the efficacy and safety of upadacitinib through 2 years.

**Methods:**

The study enrolled eligible adult patients with a clinical diagnosis of nr-axSpA who met the 2009 Assessment of SpondyloArthritis international Society (ASAS) classification criteria and had objective signs of active inflammation on magnetic resonance imaging (MRI) of sacroiliac joints and/or high-sensitivity C-reactive protein. Patients were randomized 1:1 to receive double-blinded treatment with upadacitinib 15 mg once daily (QD) or placebo for 52 weeks, after which all patients received open-label treatment with upadacitinib 15 mg QD. Efficacy results over 104 weeks were reported as observed (AO) and either AO with non-responder imputation (AO-NRI; binary endpoints) or AO with mixed-effect model for repeated measures (continuous endpoints). Treatment-emergent adverse events (TEAEs) were summarized through week 104.

**Results:**

Of 313 patients randomized and treated, 224 (continuous upadacitinib *n* = 117; placebo/upadacitinib *n* = 107) completed 104 weeks of treatment. In patients who received continuous upadacitinib, sustained improvement was observed through 2 years of treatment across efficacy endpoints including disease activity, pain, function, enthesitis, quality of life, and MRI measures of inflammation. At week 104, 57.1%, 59.0%, and 31.4% of patients achieved ASAS40 response, and low disease activity and inactive disease (as defined by Axial Spondyloarthritis Disease Activity Score), respectively (AO-NRI); week 104 outcomes were generally similar in patients who initially received placebo and were switched to upadacitinib at week 52. In total, 286 patients were exposed to ≥ 1 dose of upadacitinib, comprising 378.3 patient-years (PY) of exposure. Upadacitinib was generally well tolerated, with exposure-adjusted event rates (EAERs) for TEAEs, serious adverse events (AEs), and AEs leading to study drug discontinuation of 207.5, 8.7, and 5.3 events/100 PY, respectively. EAERs of TEAEs of special interest were broadly consistent with those reported through week 52.

**Conclusions:**

Treatment with upadacitinib demonstrated consistent improvement and maintenance of treatment effect across efficacy endpoints through 2 years; no new safety signals were identified with additional exposure.

**Trial registration:**

NCT04169373.

**Supplementary Information:**

The online version contains supplementary material available at 10.1186/s13075-024-03441-3.

## Background

Non-radiographic axial spondyloarthritis (nr-axSpA) is characterized by inflammation in the sacroiliac (SI) joints and the spine [1]. Compared with patients with radiographic axSpA (also known as ankylosing spondylitis [AS]), patients with nr-axSpA experience a similar burden of back pain, functional impairment, and impact on quality of life (QoL), and greater prevalence of peripheral involvement, even though they may not have definite sacroiliitis on radiographs [2].

Assessment of SpondyloArthritis international Society (ASAS)/European Alliance of Associations for Rheumatology (EULAR) recommendations advise first-line pharmacologic treatment with non-steroidal anti-inflammatory drugs (NSAIDs) for axSpA [3]. Patients with inadequate response to NSAIDs and predominantly axial disease with persistent disease activity are recommended for treatment with a biologic disease-modifying antirheumatic drug (bDMARD) or Janus kinase (JAK) inhibitor [3].

Upadacitinib is an oral, reversible, and selective JAK inhibitor that has demonstrated efficacy and an acceptable safety profile in axSpA and other inflammatory conditions, including psoriatic arthritis and rheumatoid arthritis, as well as inflammatory bowel disease and atopic dermatitis [4–10]. In the phase 3 SELECT-AXIS 2 program (NCT04169373), two individual placebo-controlled studies were conducted to evaluate upadacitinib 15 mg once daily (QD) in patients with axSpA: one study in patients with AS and prior inadequate response to bDMARDs, and a second study in patients with nr-axSpA who were bDMARD-naïve or had an inadequate response to prior bDMARD treatment [4, 5, 11, 12]. In both studies, treatment with upadacitinib was associated with significant improvements in the signs and symptoms of axSpA (both AS and nr-axSpA) versus placebo and was well tolerated through 14 weeks of treatment; improvements versus placebo were sustained through 1 year in the nr-axSpA study [4, 5, 12], with a safety profile consistent with that reported across indications [6–8].

Here, we report the efficacy and safety of upadacitinib 15 mg QD through 2 years of treatment in the SELECT-AXIS 2 nr-axSpA study.

## Methods

### Study design and treatment

SELECT-AXIS 2 nr-axSpA (NCT04169373) was a 1-year randomized, double-blind, placebo-controlled, multi-center trial, the methodology of which has been described previously [4, 12]. Patients enrolled from November 2019 were randomly assigned to receive upadacitinib 15 mg QD or placebo during a 52-week, double-blind treatment period, the duration of which was chosen to accommodate regulatory requirements by the US Food and Drug Administration. All patients then received upadacitinib 15 mg QD in an open-label extension to week 104 (last patient visit date June 2023).

Through week 52, stable doses of background medications were allowed, including conventional synthetic DMARDs (csDMARDs), oral corticosteroids, and NSAIDs. Changes to these background medications were allowed after week 52 per the investigator’s discretion.

### Patients

Adults aged 18 years or older who had a clinical diagnosis of nr-axSpA and met the 2009 ASAS classification criteria as assessed by the investigator and did not have radiographic sacroiliitis at baseline were eligible to participate in the SELECT-AXIS 2 nr-axSpA study [4, 12]. At screening, magnetic resonance imaging (MRI) of SI joints was performed and centrally read, and high-sensitivity C-reactive protein (hsCRP) was centrally analyzed to identify objective signs of active inflammation for study eligibility. Additional eligibility criteria included active disease as defined by the Bath Ankylosing Spondylitis Disease Activity Index (BASDAI), the patient’s assessment of total back pain scores of ≥ 4, and a prior inadequate response to ≥ 2 NSAIDs, or intolerance to or contraindication for NSAIDs. Patients with a history of inflammatory arthritis other than axSpA, those with prior exposure to a JAK inhibitor, those meeting the radiographic criterion of the modified New York criteria [4, 12] (based on pelvic radiographs of the SI joint obtained at screening), or those with active fibromyalgia were excluded.

Per protocol, 20–35% of patients were required to have had previous treatment with one bDMARD (a tumor necrosis factor [TNF] or interleukin [IL]-17 inhibitor) and to have discontinued due to either lack of efficacy or intolerance.

### Rescue criteria

Patients who did not achieve relative improvement of at least 20% in three out of the four ASAS domains without worsening in the remaining domain (ASAS20), at two consecutive study visits between week 24 and week 52, met rescue criteria and were eligible for rescue therapy per the investigator’s discretion based on local standard of care. Patients who initiated rescue therapy could continue study drug treatment unless the rescue therapy was a bDMARD, in which case the study drug was permanently discontinued. Concomitant axSpA medications (e.g., csDMARDs, oral corticosteroids, and NSAIDs) could be adjusted at the investigator’s discretion for patients meeting rescue criteria, or for any patient (regardless of disease activity status) from week 52 onward.

The study was conducted according to the International Council for Harmonisation guidelines, local regulations and guidelines governing clinical study conduct, and the Declaration of Helsinki. All patients provided written informed consent, and the study protocol and consent forms were approved by an institutional review board or independent ethics committee at each study site.

### Assessments

#### Efficacy endpoints

Pre-specified efficacy endpoints were evaluated through week 104 and included the proportion of patients achieving ≥ 40% improvement in three out of the four ASAS domains without worsening in the remaining domain (ASAS40), Axial Spondyloarthritis Disease Activity Score low disease activity (ASDAS LDA; < 2.1), ASDAS inactive disease (< 1.3), ASDAS major improvement; ≥ 2-point decrease from baseline), ASDAS clinically important improvement (CII; ≥ 1.1-point decrease from baseline) [13, 14], and ASAS partial remission. Additional pre-specified efficacy endpoints included ≥ 50% improvement from baseline in BASDAI (BASDAI50); change from baseline in ASDAS, fatigue/tiredness (BASDAI question 1; see also Table S1), patient assessment of total back pain and nocturnal back pain, severity and duration of morning stiffness (BASDAI questions 5 and 6, respectively), Bath Ankylosing Spondylitis Functional Index (BASFI), Ankylosing Spondylitis Quality of Life (ASQoL), ASAS Health Index (HI), linear Bath Ankylosing Spondylitis Metrology Index (BASMI [15]), and Maastricht Ankylosing Spondylitis Enthesitis Score in patients presenting with baseline enthesitis. In addition, individual ASAS and ASDAS components, and tender and swollen joint counts were recorded.

MRIs of SI joints and the spine were performed at baseline, week 14, and week 104. Lateral radiographs of the cervical and lumbar spine were obtained at screening and week 104. For patients who had available image(s) beyond week 14, all images collected up to week 104 were read centrally. In brief, reading and interpretation of imaging data were conducted by two independent reviewers with expertise in musculoskeletal imaging and axSpA; a third reviewer was assigned to adjudicate discrepancies between the readers exceeding a certain threshold (full details, including methodology for score calculation, have been published previously [4]; the thresholds used for this analysis can be found in the supplementary methods). Screening, week 14, and week 104 images were read together in a single reading session. Reviewers were blinded to timepoint, initial treatment assignment, subject, and site-identifying information. Mean change from baseline was calculated from the two primary reviewers, or from the two closest scores if a third reading was performed by an adjudicator. Analyses included change from baseline in MRI Spondyloarthritis Research Consortium of Canada (SPARCC) scores of the SI joints and spine [16, 17] and change from baseline in modified Stoke Ankylosing Spondylitis Spinal Score (mSASSS) on lateral radiographs of the lumbar and cervical spine [18].

#### Safety endpoints

Treatment-emergent adverse events were recorded from the date of the first dose of study drug up to week 104 (with 30-day follow-up for patients who exited the trial at or prior to week 104) and were coded using the Medical Dictionary for Regulatory Activities version 26.0. Blinded evaluation of all major adverse cardiovascular events (MACE) and venous thromboembolic events (VTE) was completed by an independent cardiovascular adjudication committee. Laboratory assessments were conducted through week 104 and are reported as the number and proportion of patients meeting criteria for potentially clinically significant values through week 104 (grade 3 or 4 based on Common Terminology Criteria for AEs [19]), as well as change from baseline in each parameter.

### Statistical analysis

#### Efficacy

Efficacy analyses were performed according to randomized treatment group for all patients who were randomized and received at least one dose of study drug. Binary endpoints are reported using as observed (AO) and AO with non-responder imputation analyses (AO-NRI, where all observed data [including after use of rescue therapy] are used and missing data are imputed as non-responders). For continuous endpoints, least squares (LS) mean change from baseline are reported from the mixed-effect model for repeated measures analysis on AO data (AO-MMRM). The model included the fixed effects of treatment, visit, treatment-by-visit interaction, and main stratification factors of screening MRI and hsCRP status, and the continuous fixed covariate of baseline measurement. ANCOVA was used for LS mean change from baseline in mSASSS at week 104; the model included treatment and screening hsCRP status as fixed factors and baseline value as a covariate. Post-hoc analysis was performed to assess radiographic progression, defined as a change from baseline in mSASSS ≥ 2. Additional post-hoc analyses were also performed for efficacy endpoints at week 104 in subgroups of patients who were bDMARD-naïve, or had received previous treatment with a bDMARD, TNF inhibitor, or an IL-17 inhibitor, and for ASAS40 response and change from baseline in hsCRP over time in patients who had elevated hsCRP (> 5 or > 7 mg/L) at baseline.

#### Safety

Safety data are reported through week 104 (with 30-day follow-up for patients who exited the trial at or prior to week 104) for all patients who received at least one dose of study drug. Exposure-adjusted event rates (events/100 patient-years [E/100 PY]) and exposure-adjusted incidence rates (*n*/100 PY) were calculated for treatment-emergent adverse events. Descriptive statistics are provided for laboratory parameters, as noted above.

## Results

### Patient disposition and characteristics

**Fig. 1:**
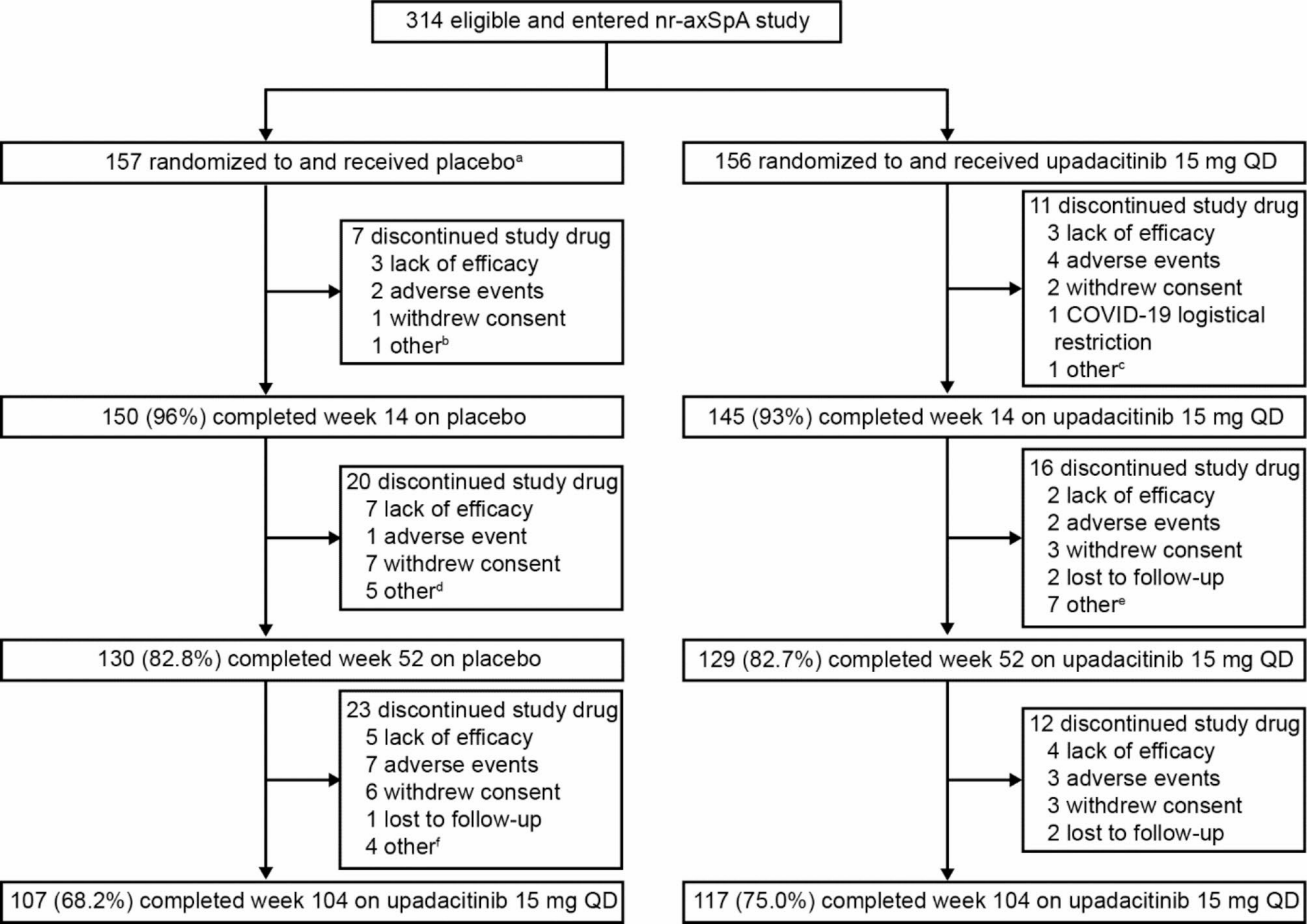
Patient disposition through week 104 Primary reason for discontinuation presented. ^a^One patient decided not to participate after randomization and discontinued the study before receiving study drug. ^b^One patient discontinued study treatment due to not meeting the inclusion criteria. ^c^One patient discontinued study treatment due to relocating geographically. ^d^Other primary reasons for discontinuation were candida supplement use (*n* = 1), antibiotic medication use (*n* = 1), early rescue with a bDMARD (*n* = 1), and patient decision (*n* = 1). ^e^Other primary reasons for discontinuation were site closure (*n* = 1), patient decision (*n* = 3), not meeting the inclusion criteria (*n* = 1), inability to follow study procedures (*n* = 1), and tuberculosis medication use (*n* = 1). ^f^Other primary reasons for discontinuation were due to moving out of the country (*n* = 2), withdrew consent (*n* = 1), and patient decision (*n* = 1). *nr-axSpA* non-radiographic axial spondyloarthritis, *QD* once daily

Of 313 patients randomized who received study drug, 259 (82.7%) entered the OLE on study drug at week 52 (continuous upadacitinib, *n* = 129; placebo/upadacitinib, *n* = 130; Fig. 1). A total of 117 patients in the continuous upadacitinib group (75.0%) and 107 patients in the placebo/upadacitinib group (68.2%) completed study drug treatment through week 104 (Fig. 1). Most discontinuations occurred before week 52 and have been previously reported [12]; 12 and 23 discontinuations occurred in the continuous upadacitinib and placebo/upadacitinib groups, respectively, in the open-label period. The most common primary reasons for discontinuation across both groups in the open-label period were AEs (*n* = 10), withdrawal of consent (*n* = 9), and lack of efficacy (*n* = 9).

Baseline demographics and characteristics, which have been reported previously in more detail [4], were comparable between treatment groups. Overall, 58.5% (*n* = 183) and 45.0% (*n* = 141) had elevated hsCRP > 5 and > 7 mg/L, respectively. At baseline, 75% of patients were receiving concomitant NSAIDs, 29% csDMARDs, and 11% oral corticosteroids. Through week 104, 79.6%, 33.9%, and 17.6% of patients had received concomitant NSAIDs, csDMARDs, and oral corticosteroids, respectively, at some point during the study. Of the 259 patients who entered the open-label period beyond week 52, 24 (9%) received new treatment or increased dose of treatment for nr-axSpA after week 52, most often NSAIDs and short-term glucocorticoids. Of patients receiving concomitant opioids at baseline (7.7%), almost half had discontinued opioids by week 104 (50.0% and 44.4% for patients receiving continuous upadacitinib and that switched from placebo to upadacitinib, respectively).

In total, 32.9% of patients had previous treatment with a bDMARD (27.8% with a TNF inhibitor and 6.1% with an IL-17 inhibitor; 1.0% of patients [*n* = 3] had previous treatment with both a TNF inhibitor and an IL-17 inhibitor).

### Efficacy

**Fig. 2:**
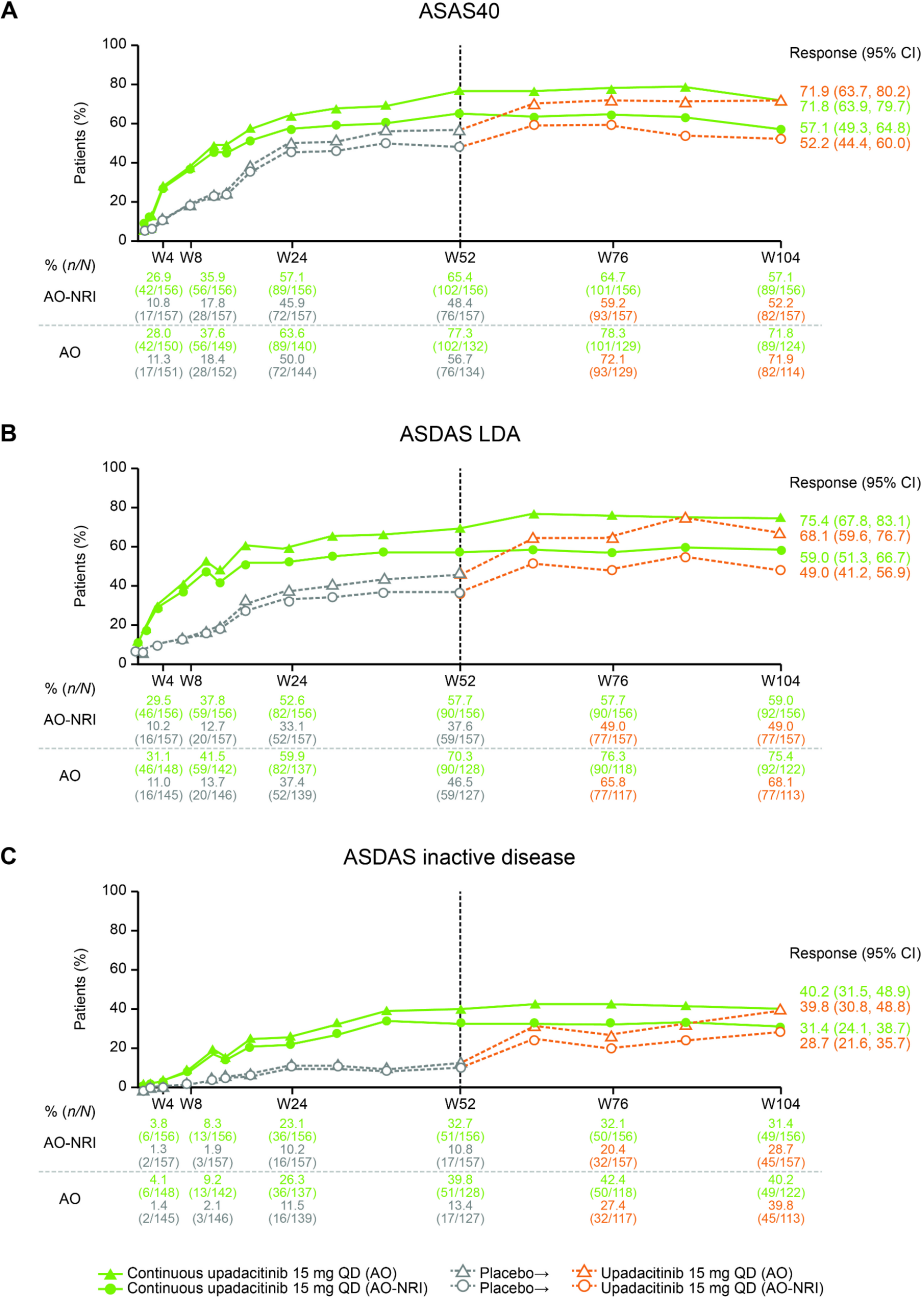
Proportion of patients achieving ASAS40, ASDAS LDA, and ASDAS inactive disease through week 104 *AO* as observed, *ASAS40* ≥ 40% improvement in three out of the four of the Assessment of SpondyloArthritis International Society domains without worsening in the remaining domain, *ASDAS* Axial Spondyloarthritis Disease Activity Score, *CI* confidence interval, *LDA* low disease activity, *MI* multiple imputation, *nr-axSpA* non-radiographic axial spondyloarthritis, *NRI* non-responder imputation, *PBO* placebo, *QD* once daily, *UPA* upadacitinib, *W* week

The proportion of patients who received continuous upadacitinib treatment who achieved ASAS40 response was generally maintained from week 52 through week 104, with 57.1% of patients achieving ASAS40 response at week 104 (AO-NRI; Fig. 2A). The proportions of patients achieving ASDAS LDA and ASDAS inactive disease followed a similar trend, with 59.0% and 31.4% of patients achieving these outcomes at week 104, respectively (AO-NRI; Fig. 2B, C).

ASDAS mean change from baseline, ASDAS major improvement, and ASDAS CII showed similar patterns of response compared with other efficacy variables (Fig. S1A–C). In patients who received continuous upadacitinib, responses were maintained through week 104, with an ASDAS mean change from baseline to week 104 of -1.9 (AO-MMRM), and 40.4% and 60.3% of patients achieving ASDAS major improvement and ASDAS CII at week 104, respectively (AO-NRI). A similar trend was observed for the proportions of patients achieving ASAS partial remission and BASDAI50 (Fig. S1D–E), with 37.2% and 56.4% of patients who received continuous upadacitinib achieving these outcomes at week 104, respectively (AO-NRI).

**Fig. 3:**
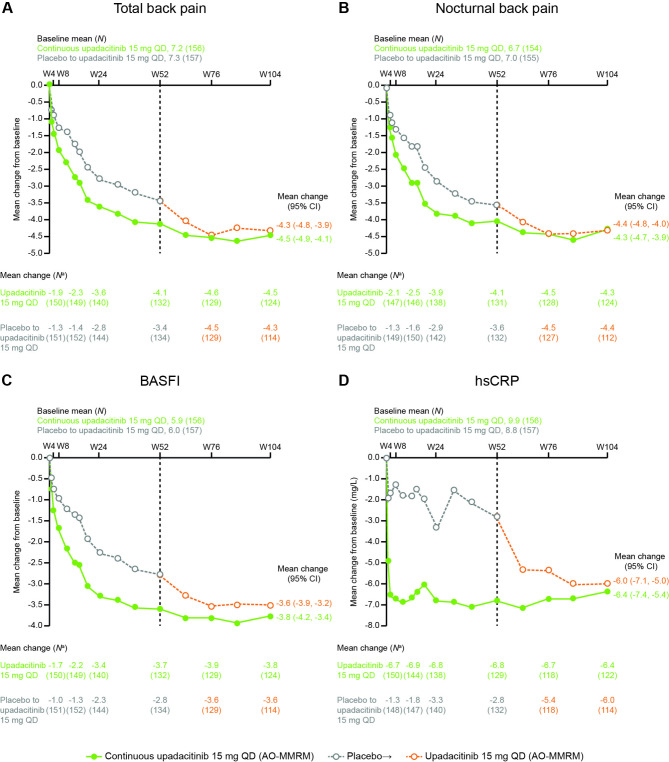
Change from baseline in total and nocturnal back pain, BASFI, and hsCRP through week 104 ^a^*N* is the number of patients with observed data at each visit *AO* as observed, *BASFI* Bath Ankylosing Spondylitis Functional Index, *CI* confidence interval, *hsCRP* high-sensitivity C-reactive protein, *MMRM* mixed-effect model for repeated measures, *PBO* placebo, *QD* once daily, *UPA* upadacitinib, *W* week

For patients who received continuous upadacitinib, reductions observed in total back pain and nocturnal back pain at week 52 were sustained through week 104 (Fig. 3A, B; mean change from baseline to week 104 = -4.5 and -4.3, respectively [AO-MMRM]). Similarly, improvements observed at week 52 in BASFI and hsCRP with continuous upadacitinib were sustained to week 104 (Fig. 3C, D; mean change from baseline to week 104 = -3.8 and − 6.4 mg/L, respectively [AO-MMRM]).

After week 52, patients who received continuous upadacitinib showed further improvements and maintained responses through week 104 in QoL (ASQoL; mean change from baseline to week 104 = -7.4 [AO-MMRM]) and overall health status (ASAS HI; mean change from baseline to week 104 = -4.9 [AO-MMRM]) (Fig. S2A, B). Treatment with continuous upadacitinib resulted in consistent improvements in peripheral musculoskeletal signs at week 104 (tender and swollen joint counts, Table 1; and MASES in patients with baseline enthesitis, Fig. S2C). Similarly, treatment with continuous upadacitinib resulted in continuous improvement in spinal mobility (BASMI) through week 104 (Fig. S2D). Similar patterns were also observed across all components of the BASDAI questionnaire (Table 1).

**Table 1:** Mean change from baseline for additional efficacy outcomes at week 104

Endpoint	LS mean change from baseline (95% CI)^a^	
	Placebo to upadacitinib 15 mg QD (*n* = 156)	Upadacitinib 15 mg QD (*n* = 154)
Patient Global Assessment of disease activity	-4.3 (-4.7, -3.9)	-4.4 (-4.8, -4.0)
Fatigue/tiredness (BASDAI Question 1)	-3.8 (-4.3, -3.4)	-4.0 (-4.4, -3.5)
Patient assessment of total back pain (BASDAI Question 2)	-4.3 (-4.8, -3.9)	-4.5 (-4.9, -4.1)
Peripheral pain/swelling (BASDAI Question 3)	-4.0 (-4.4, -3.5)	-4.0 (-4.4, -3.6)
Tenderness (BASDAI Question 4)	-4.0 (-4.5, -3.6)	-4.3 (-4.7, -3.9)
Severity of morning stiffness (BASDAI Question 5)	-4.5 (-4.9, -4.1)	-4.6, (-5.0, -4.2)
Duration of morning stiffness (BASDAI Question 6)	-3.8 (-4.2, -3.4)	-3.9 (-4.3, -3.5)
Mean of morning stiffness severity and duration (Mean of BASDAI Questions 5 and 6)	-4.1 (-4.5, -3.7)	-4.2 (-4.6, -3.8)
TJC68	-5.6 (-6.3, -5.0)^b^	-5.8 (-6.5, -5.1)^c^
SJC66	-1.7 (-2.0, -1.4)^b^	-1.9 (-2.2, -1.7)^c^

^a^Data are reported AO-MMRM, with *n* as the number of patients contributing to the MMRM estimate. ^b^*n* = 151. ^c^*n* = 147

*BASDAI* Bath Ankylosing Spondylitis Disease Activity Index, *CI* confidence interval, *LS* least squares, *MMRM* mixed-effect model for repeated measures, *QD* once daily, SJC66 swollen joint count out of 66 joints (among patients with baseline SJC > 0); TJC68 tender joint count out of 68 joints (among patients with baseline TJC > 0)

Of patients who were initially randomized to placebo and then switched to upadacitinib at week 52, efficacy responses rapidly increased after week 52; at week 104, responses were generally of similar magnitude to that observed for patients who received continuous upadacitinib. However, ASDAS LDA response remained numerically slightly lower for patients who initially started on placebo (Fig. 2B).

Improvements in active inflammation observed from baseline to week 14 in MRI SPARCC scores of the SI joints and spine [4] were maintained through week 104 in patients who received continuous upadacitinib (Table 2; at week 104: -2.3 [baseline mean 5.1] and − 0.7 [baseline mean 2.1; both AO-MMRM]). LS mean change from baseline to week 104 in mSASSS score was 0.00 for the continuous upadacitinib group (AO); none of the patients in this group showed mSASSS progression of ≥ 2 mSASSS points. Only one patient in the placebo to upadacitinib group showed mSASSS progression of ≥ 2 mSASSS points.

**Table 2:** Summary of imaging outcomes at week 104

Outcomes	Placebo to upadacitinib 15 mg QD (*n* = 121)	Upadacitinib 15 mg QD (*n* = 122)
No radiographic progression, % (95% CI)^a, b^	99.1 (97.4, 100.0)^e^	100.0 (100.0, 100.0)^f^
CFB in mSASSS. LS mean (95% CI)^c^	0.0 (0.0, 0.1)^e^	0.0 (-0.1, 0.1)^f^
CFB in MRI SPARCC score of SI joints, mean (95% CI)^d^	-2.3 (-3.2, -1.5)	-2.3 (-3.1, -1.5)^g^
CFB in MRI SPARCC score of spine, mean (95% CI)^d^	-0.5 (-1.2, 0.2)	-0.7 (-1.4, 0.0)

^a^Data are reported AO. ^b^Defined as CFB in mSASSS < 2. ^c^Data are reported AO with LS mean change from baseline and 95% CI using the ANCOVA model. ^d^Data are reported AO-MMRM, with *n* as the number of patients contributing to the MMRM estimate. ^e^*n* = 113. ^f^*n* = 118. ^g^*n* = 126

*AO* as observed, *CI* confidence interval, *CFB* change from baseline, *LS* least squares, *MMRM* mixed-effect model for repeated measures, *MRI* magnetic resonance imaging, *mSASSS* modified Stoke Ankylosing Spondylitis Spinal Score, *QD* once daily, *SI* sacroiliac, *SPARCC* Spondyloarthritis Research Consortium of Canada

In the subgroup analysis of patients with elevated hsCRP at baseline, patients who received continuous upadacitinib experienced rapid improvement (reduction) in hsCRP through the first 4 weeks of treatment; patients with a baseline hsCRP > 5 mg/L and > 7 mg/mL experienced a mean change from baseline of -5.04 and − 5.91, respectively, and maintained an overall reduction in hsCRP level through week 104 (Fig. S3A, B). In contrast, patients who received placebo generally experienced no improvement, and potentially some worsening (elevation) in hsCRP through week 52, although this rapidly improved upon switching to upadacitinib treatment after week 52 (Fig. S3A, B). Clinical outcome measures, such as ASAS40, also improved rapidly through the first 4 weeks of treatment with continuous upadacitinib in patients with elevated hsCRP at baseline (Fig. S3C, D).

In the subgroups of patients who had previous treatment with a bDMARD (*n* = 103; TNF inhibitor only [*n* = 84], IL-17 inhibitor only [*n* = 16], or TNF inhibitor and IL-17 inhibitor only [*n* = 3]) response rates were consistent overall, but slightly numerically lower versus the total population for ASAS40 and other efficacy endpoints at week 104 in both the continuous upadacitinib and placebo to upadacitinib groups (Table S2).

### Safety

Through week 104, 286 patients were exposed to ≥ 1 dose of upadacitinib 15 mg QD, comprising 378.3 PY of exposure. A total of 785 treatment-emergent adverse events were reported over 104 weeks in patients receiving upadacitinib (exposure-adjusted event rate 207.5 E/100 PY), of which 33 were serious (8.7 E/100 PY), and 20 led to discontinuation of study drug (5.3 E/100 PY). The most frequently reported treatment-emergent adverse events (≥ 5 E/100 PY) through week 104 were COVID-19 (78 events; 20.6 E/100 PY), nasopharyngitis (30 events; 7.9 E/100 PY), headache (28 events; 7.4 E/100 PY), hypertension (19 events; 5.0 E/100 PY), and urinary tract infection (19 events; 5.0 E/100 PY). Overall, exposure-adjusted incidence rates were similar to exposure-adjusted event rates (Fig. S4), although exposure-adjusted incidence rates of hepatic disorders were lower than those reported in the exposure-adjusted event rates.

**Fig. 4:**
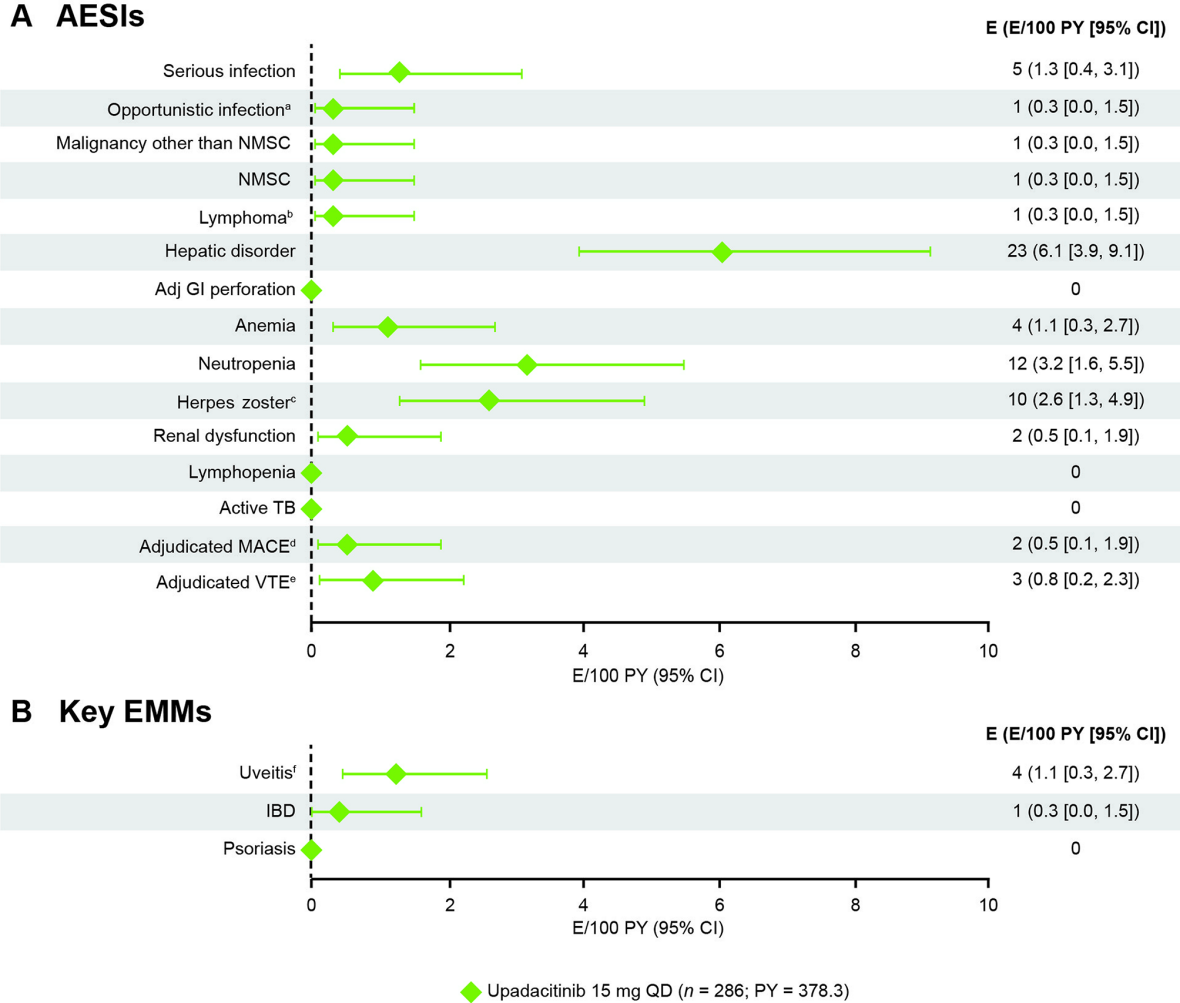
Exposure-adjusted event rates of AESIs and key EMMs through week 104 ^a^Excluding TB and herpes zoster. ^b^Adverse event of atypical lymphocytes (transient laboratory phenomenon; not true lymphoma). ^c^No serious events of herpes zoster were reported. ^d^Defined as cardiovascular death, non-fatal myocardial infarction and non-fatal stroke. There was one event of non-fatal stroke and one event of non-fatal myocardial infarction. ^e^Includes DVT and PE. There was one event of DVT and two events of PE. ^f^Includes uveitis, iritis, and iridocyclitis. *AESI* adverse event of special interest, *Adj* adjudicated, *CI* confidence interval, *DVT* deep vein thrombosis, *E* event, *EMM* Extra-musculoskeletal manifestation, *IBD* inflammatory bowel disease, *NMSC* non-melanoma skin cancer, *PE* pulmonary embolism, *PY* patient-years, *QD* once daily, *TB* tuberculosis, *UPA* upadacitinib, *VTE* venous thromboembolism

The most frequently reported AEs of special interest were hepatic disorders (23 events; 6.1 E/100 PY) and neutropenia (12 events; 3.2 E/100 PY; Fig. 4A). All hepatic disorder and neutropenia events were non-serious; most reports of hepatic disorder were transient increases of aminotransferases, and there were no cases of drug-induced liver injury per Hy’s law criteria identified [20]. No events of neutropenia led to permanent discontinuation of upadacitinib, however, one patient discontinued upadacitinib due to metabolic-associated fatty liver disease in stage F4. Similarly, anemia events (4 events; 1.1 E/100 PY) were non-serious and did not lead to discontinuation of upadacitinib. Through week 104, there were two events of acute kidney injury (0.5 E/100 PY) reported, one of which was serious and led to hospitalization. For this event, it was determined by the investigator that there was no reasonable possibility of the event being related to upadacitinib. No events of adjudicated gastrointestinal perforation, lymphopenia, or active tuberculosis were reported through week 104.

There were five events of serious infection (1.3 E/100 PY) and one of opportunistic infection (0.3 E/100 PY; Fig. 4A). Ten events of herpes zoster infection were reported (2.6 E/100 PY), all of which were considered non-serious, mild, or moderate, and did not lead to discontinuation of upadacitinib.

Through week 104, two events of adjudicated MACE were reported (0.5 E/100 PY; Fig. 4A); one event of non-fatal stroke and one additional event of non-fatal myocardial infarction. There were three adjudicated VTE events through week 104 (0.8 E/100 PY), two of which were severe events of pulmonary embolism and one of which was a severe event of deep vein thrombosis. One event each of malignancy (excluding non-melanoma skin cancer) and non-melanoma skin cancer was reported (both 0.3 E/100 PY) through week 104. Additional safety information for key AESIs is presented in Table S3.

There were four events of uveitis (1.1 E/100 PY; three occurred with no prior history), one event of inflammatory bowel disease (0.3 E/100 PY; no prior history), and no events of psoriasis (0 E/100 PY) reported through week 104; the corresponding exposure-adjusted incidence rates were 0.8, 0.3, and 0 *n*/100 PY, respectively (Fig. 4B, Fig. S4).

### Laboratory parameters

Laboratory abnormalities of any grade were reported in a small number of patients (grade 3 and 4 changes are reported in Table S4). Across grade 3 and 4 changes in laboratory parameters, grade 3 decreases in neutrophils were the most common (reported in seven patients). Neutrophil decreases were not associated with an increased risk of serious infection, and most events were transient and returned to baseline/grade 0 values within 1 week to 5 months without permanent discontinuation of the study drug. Other grade 3 or 4 changes were reported in ≤ 2 patients. Small decreases from baseline in mean levels of hemoglobin were observed with upadacitinib treatment to week 104, with the largest decrease observed in the placebo to upadacitinib group. For both treatment groups, mean lymphocyte and neutrophil levels remained generally consistent through week 104, while small mean increases were observed in liver transaminases and creatinine with upadacitinib treatment. No cases of neutrophil, hemoglobin, or lymphocyte decrease led to study drug discontinuation.

## Discussion

Here, we report the efficacy and safety of upadacitinib 15 mg QD through 2 years in patients with nr-axSpA from the SELECT-AXIS 2 study, one of the largest phase 3 trials conducted in nr-axSpA to date and the first to investigate the use of a JAK inhibitor for this indication. Results demonstrate maintenance or further improvement of efficacy of upadacitinib from week 52 through week 104 across efficacy outcomes, including measures of disease activity, patient-reported outcomes, and objective measures of inflammation based on hsCRP and MRI. Upadacitinib was generally well tolerated through week 104 and no new safety signals were identified.\n

Sustained improvement was observed through 2 years of treatment across a comprehensive set of efficacy measures in patients who received continuous upadacitinib. Disease activity outcomes with upadacitinib in the current analysis were similar to those observed in the 1-year placebo-controlled nr-axSpA trials C-axSpAnd, COAST-X, and PREVENT (with the TNF inhibitor certolizumab pegol and IL-17 inhibitors ixekizumab and secukinumab, respectively); efficacy was similarly sustained beyond 52 weeks [21–24]. Additionally, improvements of objective inflammation on MRI observed with upadacitinib through 2 years of treatment were consistent with those observed for secukinumab in the PREVENT study, with reduced inflammation of the SI joints, and a small reduction in inflammation of the spine, observed after 2 years [25], as expected for this population.

For patients who switched from placebo to upadacitinib at week 52 in the present analysis, overall similar improvements were observed across several efficacy measures through 2 years (i.e., after 52 weeks’ upadacitinib treatment) compared with those patients who had received 2 years’ continuous upadacitinib. Similarly, in the aforementioned nr-axSpA trials, patients who were switched to active bDMARD treatment following placebo generally achieved results similar to those receiving continuous active drug [21, 23]. In some cases, there were small numeric differences, such as ASAS40 in TNF inhibitor-naïve patients, in which the placebo-switch data remained numerically lower [23]. Of note, the current study included a considerable proportion of treatment-refractory patients, with 33% of patients having a prior inadequate response to bDMARDs compared with 10% and 6% of patients having prior TNF inhibitor exposure from the PREVENT and C-axSpAnd studies, respectively [23, 24]. Although there were some improvements in patient-reported outcomes in the placebo group through week 52, a sub-analysis of patients with elevated hsCRP at baseline clearly showed evidence for persistent objective inflammation in patients who received placebo. In contrast, clear and rapid improvements in hsCRP were observed in patients treated with upadacitinib.

Although radiographic progression is a concern in the management of axSpA as it can lead to irreversible structural damage and functional impairment [26], rates of such progression are generally low in patients with nr-axSpA. Recent studies have shown that between 5% [27] and 16% of patients [28] with nr-axSpA had radiographic progression to AS within 5 years. Similarly, most patients from the PREVENT study of secukinumab in patients with nr-axSpA had no radiographic progression in the spine and SI joints over 2 years [25], and only 2% of patients with nr-axSpA in the SPEED-2 retrospective observational cohort study progressed to AS in the first 2 years [29]. Consistent with this, there was no evidence of spinal radiographic progression in patients treated with upadacitinib in this 2-year trial.

As expected from their treatment-refractory nature, efficacy outcomes at week 104 were slightly numerically lower in subgroups of patients who had prior exposure to treatment with a bDMARD (TNF inhibitor or IL-17 inhibitor) versus the total population, although low patient numbers limit interpretation of results observed in the IL-17 inhibitor subgroup. Among patients with prior exposure to bDMARD treatment in the current analysis, responses after 2 years of upadacitinib treatment showed similar trends, although were numerically lower, versus those observed in patients with active bDMARD-inadequate response AS [30]. A lower treatment effect in patients with prior exposure to bDMARD treatment versus bDMARD-naïve patients is to be expected because patients with an inadequate response to bDMARD treatment have previously not responded to treatment with a TNF inhibitor or IL-17 inhibitor. Furthermore, the subgroup of patients with an inadequate response to bDMARDs comprised patients who were less likely to be responders, i.e. were older, had longer duration of disease, lower baseline CRP, and were more likely to be female and have a history of smoking than bDMARD-naïve patients [4]. Of patients with prior exposure to a TNF inhibitor in the current analysis, ASAS40 responses with upadacitinib in patients with nr-axSpA were generally similar to those observed at 2 years of treatment with ixekizumab in TNF inhibitor-experienced patients with radiographic axSpA (ASAS40 responses: 50.0%/59.5% [AO-NRI/AO] with upadacitinib at week 104 versus 47.0%/48.5% [NRI/AO] with ixekizumab at week 116 [21]).

The long-term safety results from this study showed that upadacitinib 15 mg QD was generally well tolerated in patients with nr-axSpA over 104 weeks, as assessed by frequency of treatment-emergent adverse events based on exposure-adjusted event rate and exposure-adjusted incidence rate, including serious AEs and AEs leading to discontinuation of study drug, as well as clinical laboratory data. Overall, long-term rates of treatment-emergent adverse events of special interest through week 104 were consistent with those reported through week 52. Similarly, the safety profile was consistent with that previously reported across long-term analyses of data from the rheumatoid arthritis, psoriatic arthritis, and AS clinical trial programs for upadacitinib [8]. COVID-19 was the most frequently reported treatment-emergent adverse event observed in this study, which can be explained by the fact that the study was conducted during the COVID-19 pandemic (November 2019–June 2023). The pattern, characteristics, and incidence of COVID-19 infections observed in patients receiving upadacitinib across indications have previously been found to be generally comparable with those observed in the general population [8]. Of note, exposure-adjusted event rates of COVID-19 were similar between upadacitinib and placebo at week 52 of this study [12]. In addition, the rates of MACE, malignancy, NMSC, and VTE remained low with long-term treatment of upadacitinib in the current analysis.

Limitations of this study include the lack of imaging data at week 52 and the absence of an active comparator. This limited the ability to observe trends in imaging data over the course of the study, and limits comparisons between outcomes with upadacitinib and other available treatments for nr-axSpA. However, a placebo comparison was available through the 1-year double-blind period, which helps contextualize the efficacy and safety of upadacitinib in patients with nr-axSpA over this period.

## Conclusion

Treatment with upadacitinib 15 mg QD demonstrated consistent improvement and maintenance of treatment effect through 2 years across measures of disease activity, pain, function, enthesitis, QoL, and MRI measures of inflammation, in patients with nr-axSpA. Patients who switched from placebo to upadacitinib at 1 year generally experienced similar responses at 2 years to those who received continuous upadacitinib. Consistent with the known long-term safety profile across indications, upadacitinib was generally well tolerated, with no new safety signals identified with additional exposure.

## Electronic supplementary material

Below is the link to the electronic supplementary material.

Supplementary Material 1

## Data Availability

AbbVie is committed to responsible data sharing regarding the clinical trials we sponsor. This includes access to anonymized, individual, and trial-level data (analysis data sets), as well as other information (e.g., protocols, clinical study reports, or analysis plans), as long as the trials are not part of an ongoing or planned regulatory submission. This includes requests for clinical trial data for unlicensed products and indications. These clinical trial data can be requested by any qualified researchers who engage in rigorous, independent scientific research, and will be provided following review and approval of a research proposal, Statistical Analysis Plan, and execution of a Data Sharing Agreement. Data requests can be submitted at any time after approval in the US and Europe and after acceptance of this manuscript for publication. The data will be accessible for 12 months, with possible extensions considered. For more information on the process or to submit a request, visit the following link: https://www.abbvieclinicaltrials.com/hcp/data-sharing.

## Abbreviations

Adj: Adjudicated
AE: Adverse event
AESI: AE of special interest
ALT: Alanine transaminase
AO: As observed
AS: Ankylosing spondylitis
ASAS: Assessment of SpondyloArthritis international Society
ASAS40: ≥ 40%Improvement in three out of the four ASAS domains without worsening in the remaining domain
ASDAS: Axial Spondyloarthritis Disease Activity Score
ASQoL: Ankylosing Spondylitis QoL
AST: Aspartate transaminase
axSpA: Axial spondyloarthritis
BASFI: Bath Ankylosing Spondylitis Functional Index
BASDAI: Bath Ankylosing Spondylitis Disease Activity Index
BASDAI50 ≥ 50%: Improvement in BASDAI
BASMI: Bath Ankylosing Spondylitis Metrology Index
bDMARD: Biologic DMARD
BL: Baseline
CFB: Change from baseline
CI: Confidence interval
CII: Clinically important improvement
csDMARD: Conventional synthetic DMARD
DMARD: Disease-modifying antirheumatic drug
DVT: Deep vein thrombosis
E: Event
EMM: Extra-musculoskeletal manifestation
EULAR: European Alliance of Associations for Rheumatology
HI: Health Index
hsCRP: High-sensitivity C-reactive protein
IBD: Inflammatory bowel disease
IL: Interleukin
JAK: Janus kinase
LDA: Low disease activity
LS: Least squares
MACE: Major adverse cardiovascular event
MASES: Maastricht Ankylosing Spondylitis Enthesitis Score
MAD: Mean absolute difference
MI: Multiple imputation
MMRM: Mixed-effects model for repeated measures
MRI: Magnetic resonance imaging
mSASSS: Modified Stoke Ankylosing Spondylitis Spinal Score
NMSC: Non-melanoma skin cancer
nr-axSpA: Non-radiographic axSpA
NRI: Non-responder imputation
NRS: Numeric rating scale
NSAID: Non-steroidal anti-inflammatory drug
OLE: Open-label extension
PBO: Placebo
PE: Pulmonary embolism
PY: Patient-years
QD: Once daily
QoL: Quality of life
SAE: Serious AE
SI: Sacroiliac
SJC: Swollen joint count
SPARCC: Spondyloarthritis Research Consortium of Canada
TB: Tuberculosis
TJC: Tender joint count
TNF: Tumor necrosis factor
ULN: Upper limit of normal
UPA: Upadacitinib
VTE: Venous thromboembolic event
W: Week
