# Supplementary material for: Upadacitinib in active non-radiographic axial spondyloarthritis: 2-year data from the phase 3 SELECT-AXIS 2 study
Source: Arthritis Res Ther. 2025 Feb 4;27:23. doi: 10.1186/s13075-024-03441-3 (PMC11792409; doi:10.1186/s13075-024-03441-3)
Supplement: Supplementary file 1 — Supplementary Material 1 [file 13075_2024_3441_MOESM1_ESM.docx]

**Supplementary Material**

**Supplementary methods**

Adjudication methods for imaging endpoints:

For MRI SPARCC assessments, two primary readers blinded to treatment assignment and imaging time points independently reviewed MRIs, and a third reader was used to adjudicate discrepancies between the primary readers if the differences in spine and SI joint SPARCC change scores exceeded a certain mean absolute difference (MAD) threshold. The MAD was calculated from the absolute difference between the two primary reviewers for each subject, per reading session, per anatomy. There was a MAD for spine and a MAD for SI joints for each reading session. Adjudication was triggered if the absolute difference between the primary readers’ change scores was greater than three times the MAD for that anatomy and reading session. Only the images that triggered adjudication underwent efficacy adjudication review.

In this study, for the reading of baseline and week 14 MRIs, the adjudication trigger was an absolute difference of ≥ 14 for MRI of the spine and ≥ 8 for MRI of the SI joints. For the reading of baseline and week 104 MRIs, the adjudication trigger was an absolute difference of ≥ 15 for MRI of the spine and ≥ 9 for MRI of the SI joints. The final mean change from baseline was calculated from the two closest scores out of the three.

For mSASSS assessments, a third reader who was not one of the two primary readers adjudicated if the follow-up change scores differed by ≥ 5 mSASSS points between the two primary readers. The final mSASSS score was calculated from the two closest readings out of the three.

**Supplementary Fig. 1** ASDAS, ASDAS MI, ASDAS CII, and ASAS PR, and BASDAI50 responses through week 104


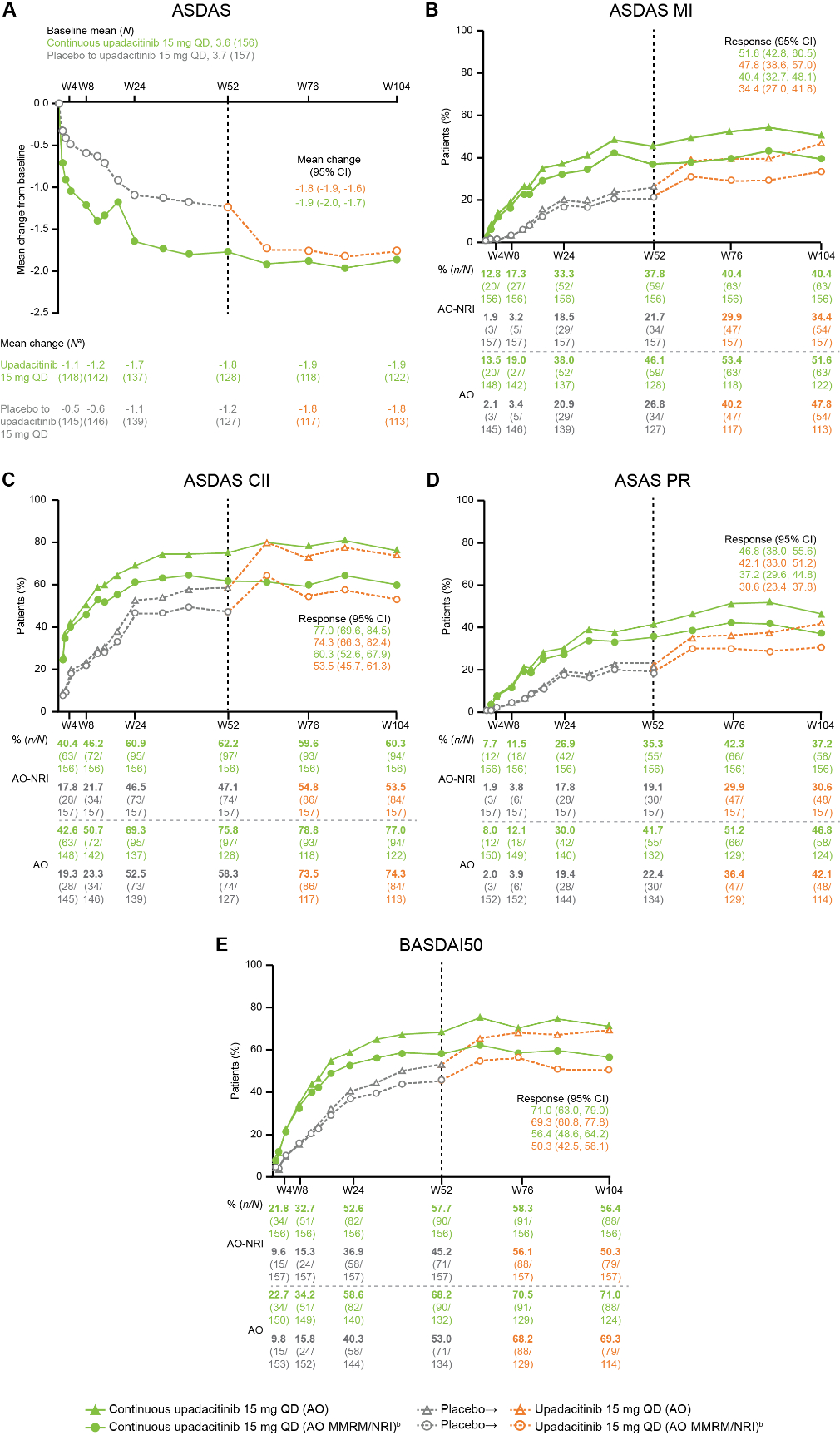


^a^*N* is the number of patients with observed data at each visit. ^b^Data are reported AO-MMRM for continuous endpoints (i.e., change from baseline in ASDAS) and AO-NRI for binary endpoints.

*AO* as observed, *ASAS* Assessment of SpondyloArthritis international Society, *ASDAS* Axial Spondyloarthritis Disease Activity Score, *BASDAI50* ≥ 50% improvement from baseline in Bath Ankylosing Spondylitis Disease Activity Index, *CI* confidence interval, *CII* clinically important improvement, *LS* least squares, *MI* major improvement, *MMRM* mixed-effect model for repeated measures, *nr-axSpA* non-radiographic axial spondyloarthritis, *NRI* non-responder imputation, *PBO* placebo, *PR* partial remission, *QD* once daily, *UPA* = upadacitinib; *W* week

**Supplementary Fig. 2** Change from baseline in ASQoL, ASAS HI, MASES, and BASMI through week 104


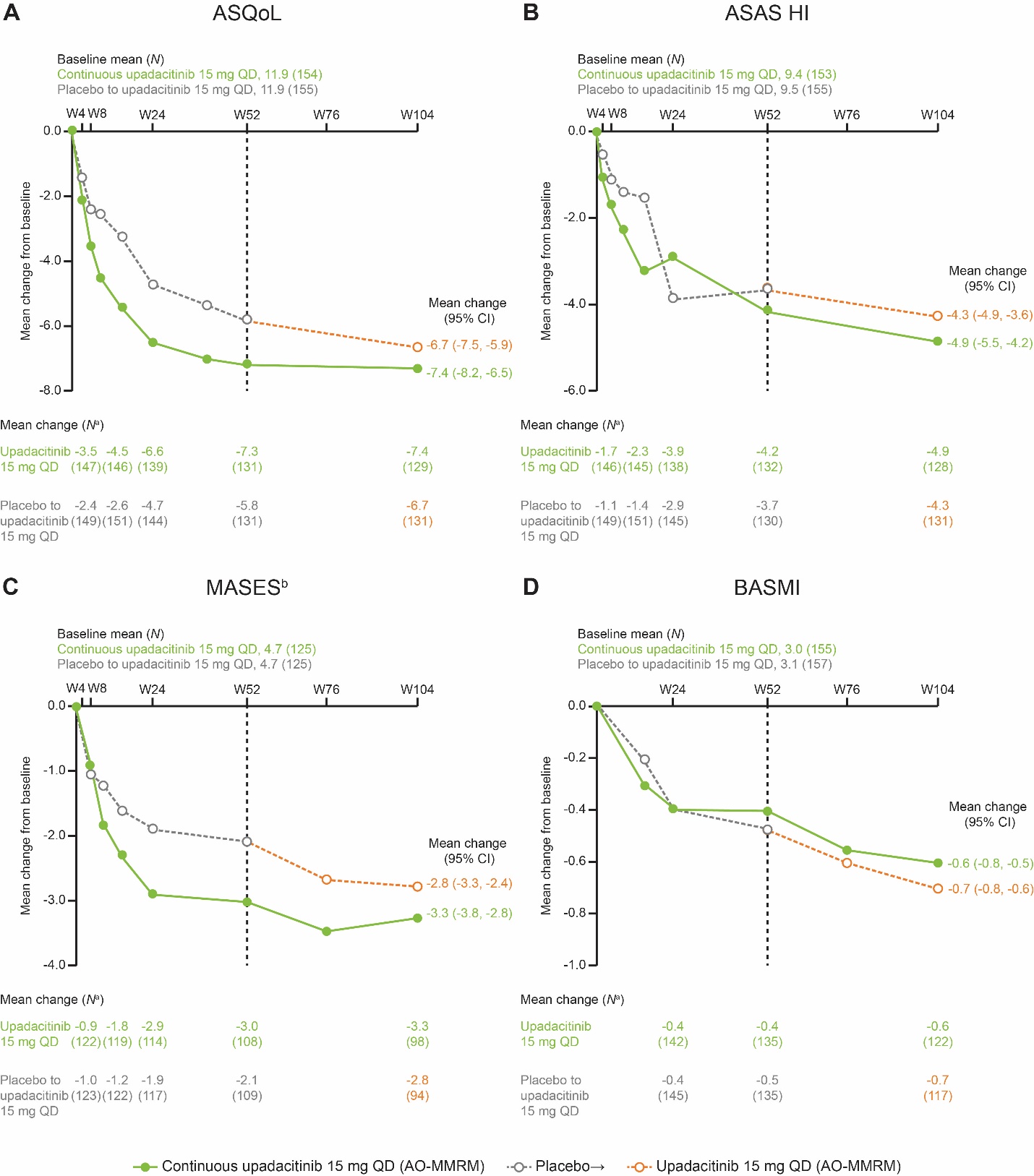


^a^*N* is the number of patients with observed data at each visit. ^b^Assessed in patients with baseline enthesitis.

*AO* as observed, *ASAS HI* Assessment of SpondyloArthritis international Society Health Index, *ASQoL* Ankylosing Spondylitis Quality of Life, *BASMI* Bath Ankylosing Spondylitis Metrology Index, *CI* confidence interval, *LS* least squares, *MASES* Maastricht Ankylosing Spondylitis Enthesitis Score, *MMRM* mixed-effect model for repeated measures, *PBO* placebo, *QD* once daily, *UPA* upadacitinib, *W* week

**Supplementary Fig. 3** hsCRP and ASAS40 responses through week 104 in patients with elevated hsCRP at baseline


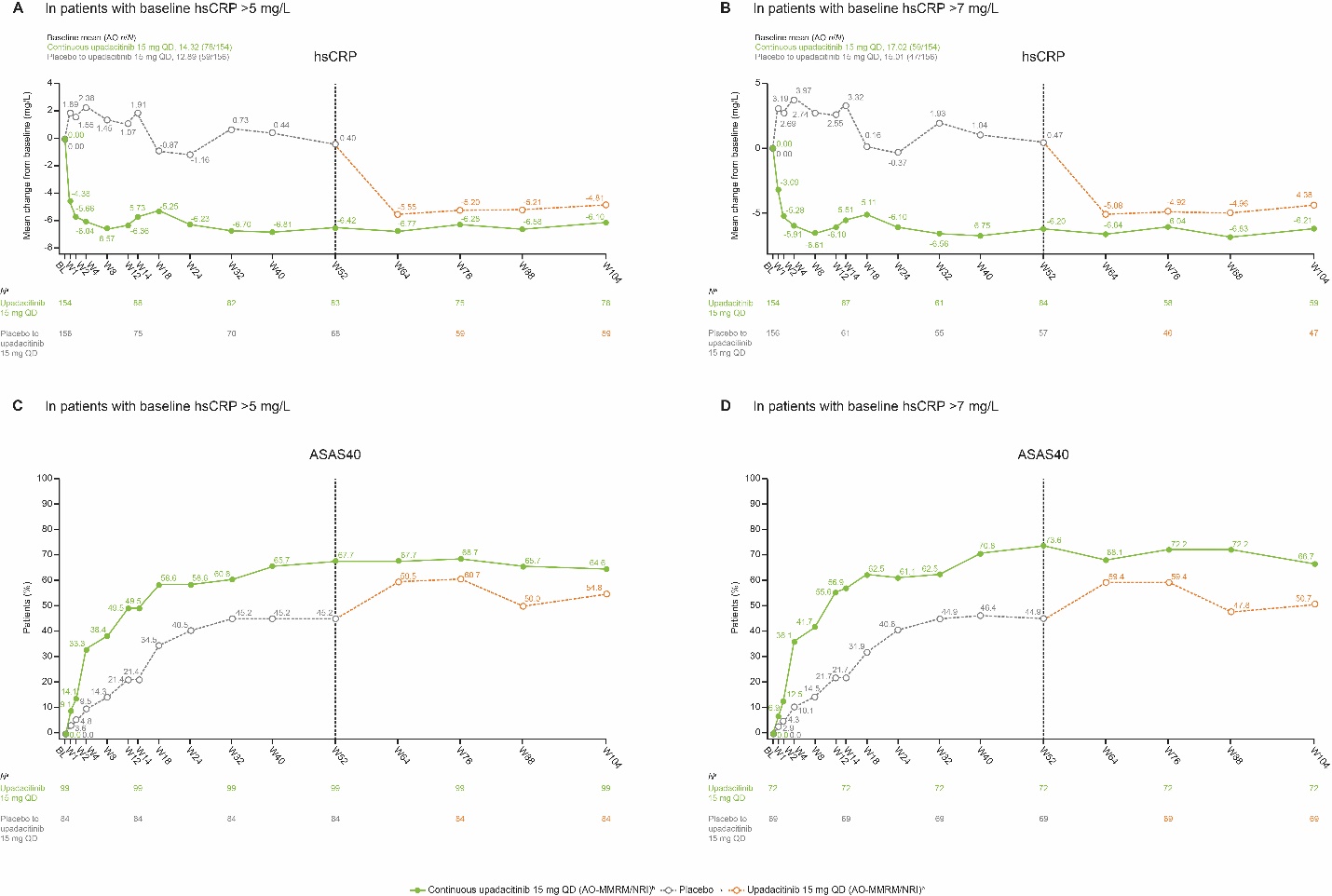


^a^*N* is the number of patients with observed data at each visit.

*AO* as observed, *ASAS40* ≥ 40% improvement in three out of the four of the Assessment of SpondyloArthritis International Society domains without worsening in the remaining domain, *hsCRP* high-sensitivity C-reactive protein, *MMRM* mixed-effect model for repeated measures, *PBO* placebo, *QD* once daily, *UPA* upadacitinib, *W* week

**Supplementary Fig. 4** EAIRs of TEAEs through week 104


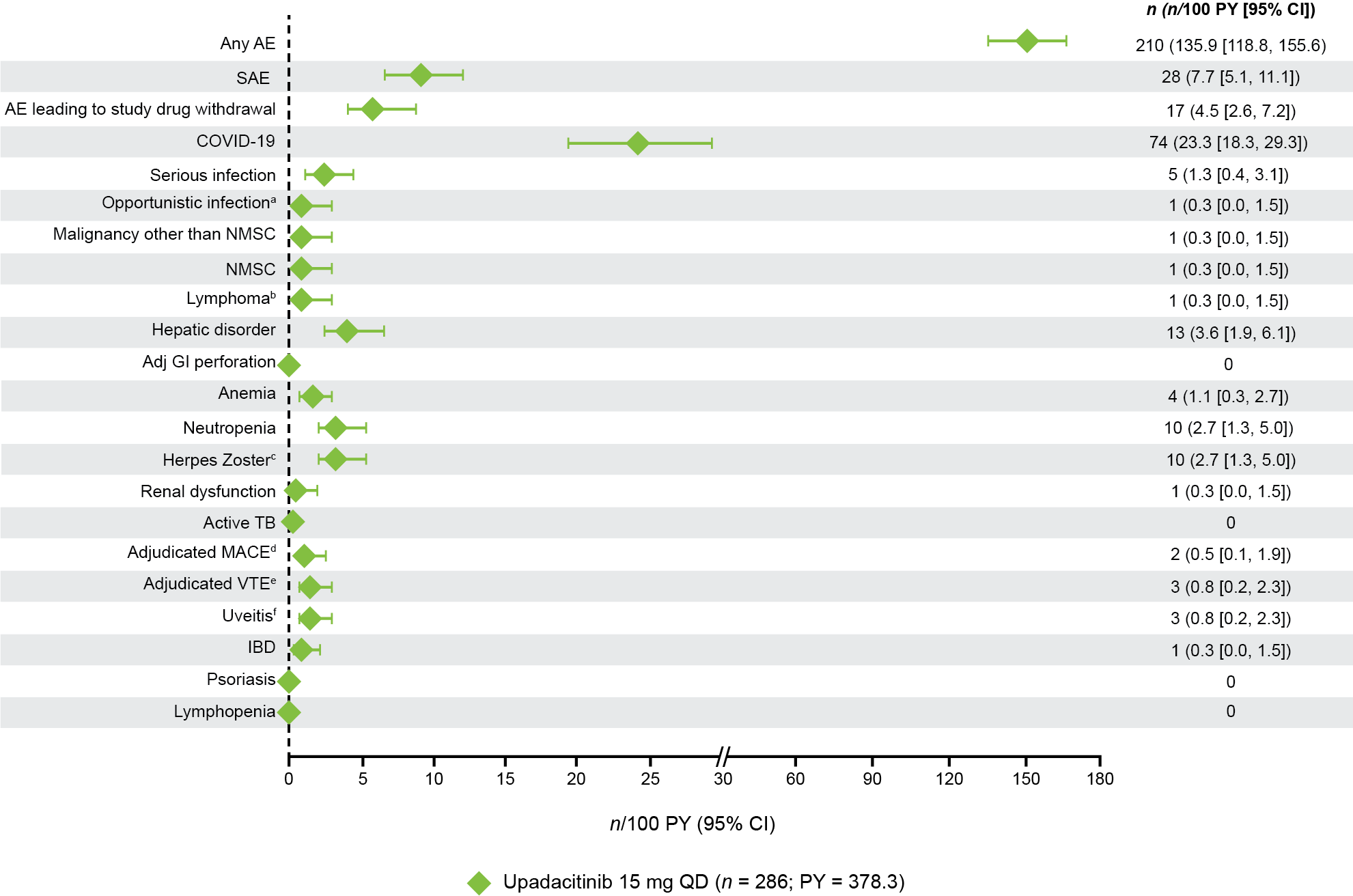


^a^Excluding TB and herpes zoster. ^b^AE of atypical lymphocytes (transient laboratory phenomenon; not true lymphoma). ^c^No serious events of herpes zoster were reported. ^d^Defined as cardiovascular death, non-fatal myocardial infarction, and non-fatal stroke. There was one event of non-fatal stroke and one event of non-fatal myocardial infarction. ^e^Includes DVT and PE. There was one event of DVT and two events of PE. ^f^Includes uveitis, iritis, and iridocyclitis.
*Adj* adjudicated, *AE* adverse event, *CI* confidence interval, *DVT* deep vein thrombosis, *E* event, *EAIR* exposure-adjusted incidence rate, *IBD* inflammatory bowel disease, *NMSC* non-melanoma skin cancer, *PE* pulmonary embolism, *PY* patient-years, *QD* once daily, *SAE* serious AE, *TB* tuberculosis, *UPA* upadacitinib, *VTE* venous thromboembolism

**Supplementary Table 1** Assessment of fatigue/tiredness, total and nocturnal back pain, and severity and duration of morning stiffness

| Outcome | Question | Scale |
| --- | --- | --- |
| Fatigue/tiredness | BASDAI question 1: *How would you describe your overall level of fatigue/tiredness?* | 0–10 NRS |
| Total back pain | *What is the amount of back pain that you experienced at any time during the last week?* | 0–10 NRS |
| Nocturnal back pain | *What is the amount of back pain at night that you experienced during the last week?* | 0–10 NRS |
| Severity of morning stiffness | BASDAI Question 5: *How would you describe your overall level of morning stiffness from the time you wake up?* | 0–10 NRS |
| Duration of morning stiffness | BASDAI Question 6: *How long does your morning stiffness last from the time you wake up?* | 0–10 NRS |

*BASDAI* Bath Ankylosing Spondylitis Disease Activity Index, *NRS* numeric rating scale

**Supplementary Table 2** Efficacy endpoints at week 104 in patients by prior treatment^a^

| **Previous treatment:** | **bDMARD-naïve** | | | | | **bDMARD** | | | | | | | **TNFi** | | | | | | **IL-17 inhibitor** | | | | |
| --- | --- | --- | --- | --- | --- | --- | --- | --- | --- | --- | --- | --- | --- | --- | --- | --- | --- | --- | --- | --- | --- | --- | --- |
| **Outcomes** | **PBO to UPA  15 mg QD** | | | **UPA  15 mg QD** | | **PBO to UPA  15 mg QD** | | | | **UPA  15 mg QD** | | | **PBO to UPA  15 mg QD** | | | **UPA  15 mg QD** | | | **PBO to UPA  15 mg QD** | | | **UPA  15 mg QD** | |
| **Patients, %** | **AO-NRI (n = 103)** | **AO (n = 74)** | **AO-NRI (n = 107)** | | **AO (n = 83)** | | **AO-NRI  (n = 54)** | **AO (n = 40)** | **AO-NRI  (n = 49)** | | **AO (n = 41)** | **AO-NRI  (n = 40)** | | **AO (n = 27)** | **AO-NRI  (n = 44)** | | **AO (n = 37)** | **AO-NRI  (n = 11)** | | **AO (n = 10)** | **AO-NRI  (n = 5)** | | **AO (n = 4)** |
| ASAS40 | 55.3 | 77.0 | 61.7 | | 79.5 | | 46.3 | 62.5 | 46.9 | | 56.1 | 45.0 | | 66.7 | 50.0 | | 59.5 | 45.5 | | 50.0 | 20.0 | | 25.0 |
| ASDAS LDA | 51.5 | 72.6^a^ | 65.4 | | 84.3 | | 44.4 | 60.0 | 44.9 | | 56.4^b^ | 42.5 | | 63.0 | 45.5 | | 57.1^c^ | 45.5 | | 50.0 | 40.0 | | 50.0 |
| ASDAS ID | 32.0 | 45.2^a^ | 40.2 | | 51.8 | | 22.2 | 30.0 | 12.2 | | 15.4^b^ | 20.0 | | 29.6 | 11.4 | | 14.3^c^ | 27.3 | | 30.0 | 20.0 | | 25.0 |
| **Mean change from baseline** | **AO-MMRM^d^** | | | | | | | | | | | | | | | | | | | | | | |
|  | **(n = 102)** | | | **(n = 105)** | | **(n = 54)** | | | | **(n = 49)** | | | **(n = 40)** | | | **(n = 44)** | | | **(n = 11)** | | | **(n = 5)** | |
| Patient’s assessment of total back pain | -4.47 | | | -4.97 | | -4.01 | | | | -3.54 | | | -4.00 | | | -3.60 | | | -4.06 | | | -2.96 | |
| Nocturnal back pain | -4.65^e^ | | | -4.81^f^ | | -3.80^g^ | | | | -3.61 | | | -3.80 | | | -3.56 | | | -3.27^h^ | | | -5.01 | |
| BASFI | -3.69 | | | -4.18 | | -3.39 | | | | -3.15 | | | -3.39 | | | -3.13 | | | -2.76 | | | -4.52 | |
| hsCRP | -6.69 | | | -6.31 | | -3.04 | | | | -4.52 | | | -2.31 | | | -4.34 | | | -4.67 | | | -7.39 | |
| Patient Global Assessment of disease activity | -4.47 | | | -5.01 | | -3.90 | | | | -3.56 | | | -3.80 | | | -3.70 | | | -3.92 | | | -3.00 | |
| Mean of morning stiffness severity and duration (mean of BASDAI Questions 5 and 6) | -4.40 | | | -4.66 | | -3.56 | | | | -3.28 | | | -3.55 | | | -3.38 | | | -2.88 | | | -3.38 | |

^a^*n* = 73.^. b^*n* = 39. ^c^ *n* = 35. ^d^*n* reported as the number of patients contributing to the MMRM estimate. ^e^ *n* = 101. ^f^*n* = 103. ^g^*n* = 53. ^h^*n* = 10.

*AO* as observed, *ASAS40* ≥40% improvement in three out of the four Assessment of SpondyloArthritis international Society domains without worsening in the remaining domain, *ASDAS* Axial Spondyloarthritis Disease Activity Score, *BASDAI* Bath Ankylosing Spondylitis Disease Activity Index, *BASFI* Bath Ankylosing Spondylitis Functional Index, *bDMARD* biologic disease-modifying antirheumatic drug, *hsCRP* high-sensitivity C-reactive protein, *ID* inactive disease, *IL* interleukin, *LDA* low disease activity, *MMRM* mixed-effect model for repeated measures, *NRI* non-responder imputation, *QD* once daily, *TNF* tumor necrosis factor

**Supplementary Table 3** Safety information for key AESIs with upadacitinib through week 104

| **AESI** | **Type** | **Age at start of study, years** | **Sex** | **Relevant medical history** | **Time to onset (days)** | **Study drug discontinuation** | **Causality^a^** |
| --- | --- | --- | --- | --- | --- | --- | --- |
| Opportunistic infection | Herpes simplex meningomyelitis | 59 | Male | Diabetes, hypertension | 675 | Yes | Reasonable possibility |
| MACE | Non-fatal stroke | 59 | Female | Dyslipidemia, former smoker | 458 | Yes | Reasonable possibility |
| MACE | Non-fatal myocardial infarction | 31 | Male | Former smoker | 680 | No | Reasonable possibility |
| VTE | PE | 43 | Female | Hypothyroidism, former smoker, developed severe PE while being hospitalized for COVID-19 pneumonia | 491 | Yes | No reasonable possibility |
| VTE | PE | 61 | Female | Hypercholesterolemia, developed severe PE after being bed bound for 2 weeks due to a back sprain | 441 | Yes | Reasonable possibility |
| VTE | DVT | 79 | Female | Fracture (bilateral arm and right wrist) | 739 | No | Reasonable possibility |
| Malignancy excl. NMSC | Invasive ductal breast cancer | 58 | Female | Previous breast atypical ductal hyperplasia, obesity | 525 | Yes | No reasonable possibility |
| NMSC | Basal cell carcinoma of the right nasal alar | 50 | Male | None recorded | 84 | No | No reasonable possibility |

^a^Relationship to study treatment as assessed by the investigator.

*AESI* adverse events of special interest, *MACE* major adverse cardiovascular event, *NMSC* non-melanoma skin cancer, *PE* pulmonary embolism, *VTE* venous thromboembolism

**Supplementary Table 4** Grade 3 or 4 laboratory parameters up to week 104

| Parameter, *n* (%) | Any upadacitinib  15 mg QD (*N* = 281) |
| --- | --- |
| Hemoglobin (g/L) |  |
| Grade 3 (< 80) | 0 |
| Lymphocytes (10^9^/L) |  |
| Grade 3 (< 0.5–0.2) | 2 (0.7) |
| Grade 4 (< 0.2) | 0 |
| Neutrophils (10^9^/L) |  |
| Grade 3 (< 1.0–0.5) | 7 (2.5) |
| Grade 4 (< 0.5) | 1 (0.4) |
| ALT (U/L) |  |
| Grade 3 (> 5.0–20.0 × ULN) | 2 (0.7) |
| Grade 4 (> 20.0 × ULN) | 0 |
| AST (U/L) |  |
| Grade 3 (> 5.0–20.0 × ULN) | 0 |
| Grade 4 (> 20.0 × ULN) | 0 |
| Serum creatinine (μmol/L) |  |
| Grade 3 (> 3.0–6.0 × ULN or > 3.0 × BL) | 1 (0.4) |
| Grade 4 (> 6.0 × ULN) | 0 |

*ALT* alanine transaminase, *AST* aspartate transaminase, *BL* baseline, *QD* once daily, *ULN* upper limit of normal
